# Clinical predictors for *Legionella *in patients presenting with community-acquired pneumonia to the emergency department

**DOI:** 10.1186/1471-2466-9-4

**Published:** 2009-01-19

**Authors:** Rico Fiumefreddo, Roya Zaborsky, Jeannine Haeuptle, Mirjam Christ-Crain, Andrej Trampuz, Ingrid Steffen, Reno Frei, Beat Müller, Philipp Schuetz

**Affiliations:** 1 Department of Internal Medicine, University Hospital Basel, Basel, Switzerland; 2 Departement of Infectious Diseases and Hospital Epidemiology, University Hospital Basel, Basel, Switzerland; 3 Institute of Medical Microbiology, University of Basel, Basel, Switzerland; 4 Department of Internal Medicine, Kantonsspital Aarau, Tellstrasse, CH-5001 Aarau, Switzerland

## Abstract

**Background:**

Legionella species cause severe forms of pneumonia with high mortality and complication rates. Accurate clinical predictors to assess the likelihood of *Legionella *community-acquired pneumonia (CAP) in patients presenting to the emergency department are lacking.

**Methods:**

We retrospectively compared clinical and laboratory data of 82 consecutive patients with *Legionella *CAP with 368 consecutive patients with non-*Legionella *CAP included in two studies at the same institution.

**Results:**

In multivariate logistic regression analysis we identified six parameters, namely high body temperature (OR 1.67, p < 0.0001), absence of sputum production (OR 3.67, p < 0.0001), low serum sodium concentrations (OR 0.89, p = 0.011), high levels of lactate dehydrogenase (OR 1.003, p = 0.007) and C-reactive protein (OR 1.006, p < 0.0001) and low platelet counts (OR 0.991, p < 0.0001), as independent predictors of *Legionella *CAP. Using optimal cut off values of these six parameters, we calculated a diagnostic score for *Legionella *CAP. The median score was significantly higher in *Legionella *CAP as compared to patients without *Legionella *(4 (IQR 3–4) vs 2 (IQR 1–2), p < 0.0001) with a respective odds ratio of 3.34 (95%CI 2.57–4.33, p < 0.0001). Receiver operating characteristics showed a high diagnostic accuracy of this diagnostic score (AUC 0.86 (95%CI 0.81–0.90), which was better as compared to each parameter alone. Of the 191 patients (42%) with a score of 0 or 1 point, only 3% had *Legionella *pneumonia. Conversely, of the 73 patients (16%) with ≥4 points, 66% of patients had *Legionella *CAP.

**Conclusion:**

Six clinical and laboratory parameters embedded in a simple diagnostic score accurately identified patients with *Legionella *CAP. If validated in future studies, this score might aid in the management of suspected *Legionella *CAP.

## Background

*Legionella *species (spp.) causes a severe form of community-acquired pneumonia (CAP) with a high incidence of adverse medical outcomes including progression of infiltrates, respiratory failure and need for intensive care unit (ICU) admission [1,2]. In addition, *Legionella *CAP has a high mortality rate of about 10 percent, which may increase up to 27 percent in patients not receiving adequate antibiotics as part of the empiric treatment on admission [2]. Early identification of *Legionella *spp. in patients presenting with respiratory symptoms and suspicion of CAP to the emergency department is thus of utmost importance because it affects the timing and choice of empirical antibiotic therapy and reduces the risk for adverse outcome. Currently available diagnostic tests include detection of *Legionella *spp. by culture or polymerase chain-reaction (PCR) in respiratory samples and *Legionella pneumophila *antigen testing in urine. These tests lack sensitivity, in addition the urine antigen test only identifies *Legionella pneumophila *serogroup 1 [3,4]. Previous studies comparing clinical, radiological and laboratory findings in *Legionella *CAP and non-*Legionella *CAP have produced controversial results [1,5-12]. Two previous attempts to construct a diagnostic score that identifies *Legionella *in patients with CAP have been disappointing [1,5-11]. As a limitation, these studies compared *Legionella pneumophila *CAP with selected cases of pneumococcal CAP [5,6,8]. When applied in unselected patients, these scores lack sensitivity and/or specificity. Because of this diagnostic dilemma consensus guidelines on empiric antibiotic therapy for patients with CAP to extend antibiotic coverage to *Legionella *in all patients with severe CAP contributing to antibiotic overuse and emergence of multi-resistant strains [13-16]. However more recent findings suggest, that severity of CAP is not an appropriate screening criterion for *Legionella *CAP, which further complicates the choice of empiric antibiotic treatment [4].

The aim of this study was to compare initial clinical and laboratory parameters of consecutive patients with *Legionella *CAP who were hospitalized in our institution during the last 10 years with patients with non-*Legionella *CAP included in two studies at the same institution [17,18] and thereby to identify reliable diagnostic predictors of *Legionella *CAP.

## Methods

### Setting and Study population

We retrospectively evaluated all consecutive patients who were admitted to the University Hospital in Basel, Switzerland from 1997 to 2007, with a diagnosis of *Legionella *CAP. The diagnosis of *Legionella *was considered definite if *Legionella *was either isolated by culture or PCR of a respiratory sample or detected by urinary antigen testing. Patient records were reviewed with a standardized data-collection form to retrieve all demographic, clinical, microbiological, radiographic, laboratory and therapeutical data. To achieve a reasonable comparison, we used clinical and laboratory data on admission to the emergency department.

For comparison we used data on admission of a consecutive cohort of 368 patients with non-*Legionella *CAP who were admitted between December 2002 through February 2005 to the same institution and enrolled in two studies [17,18]. The design of the two studies was similar and has been reported in detail elsewhere [17,18]. In brief, a total of 373 consecutive patients with radiologically proven CAP were randomly assigned to be treated either with a procalcitonin (PCT)-based algorithm or standard practice. The primary endpoint of the two studies was to evaluate antibiotic exposure of a PCT-guided treatment algorithm as compared to standard recommended guidelines [14]. Both studies excluded patients with cystic fibrosis or active pulmonary tuberculosis, hospital-acquired pneumonia and severely immunocompromized patients. In both studies, testing for *Legionella *with the use of the urine antigen test was recommended in all CAP patients as part of the hospital work up for CAP and part of the study protocols. Investigation for other atypical bacterial pathogens namely *Mycoplasma *spp. and *Chlamydia *spp. were only tested in suspected cases upon the decision of to the treating physicians. For the purpose of this study, the five enrolled patients with *Legionella *CAP were analyzed in the *Legionella *CAP group.

The two intervention studies were previously approved by the local Ethical Committee (EKBB, Ethikkommission beider Basel) and registered in the current controlled trials databases. The Institutional Review Board classified this retrospective analysis as a quality control study and waived the need for informed consent.

CAP was defined as the presence of an infiltrate on chest radiograph and at least one of the following signs and symptoms (cough, sputum production, dyspnea, core body temperature >38.0°C, auscultatory findings of abnormal breath sounds and rales [19]. In all patients the Pneumonia Severity Index (PSI) and the CURB65 were calculated based on patients unique set of variables as described in detail elsewhere [20,21]. The goldstandard for diagnosis of *Legionella *CAP was the clinical diagnosis of a community-acquired pneumonia with an infiltrate on chest X-ray and at least one positive microbiological test for *Legionella *(urinary antigen testing, culture results from respiratory specimen or PCR from respiratory specimen).

### Laboratory assessment

For our analysis, we used laboratory results including markers of infection (white blood count (WBC) and C-reactive protein (CRP)), liver enzymes (ALAT, ASAT, γ-glutamyltranspeptidase, alkaline phosphatase, bilirubin), markers of renal function (creatinine, urea), electrolytes (sodium, potassium), urinary testing for proteinuria and hemoglobinuria and results from arterial blood gas analysis (pH, PaO2) from the routinely collected blood analysis of all patients on admission. In our hospital, CRP concentrations are determined by an enzyme immunoassay having a detection limit of <5 mg/dl (EMIT, Merck Diagnostica, Zurich, Switzerland).

In the group of *Legionella *CAP *Legionella *spp. was diagnosed either by antigen in urine detection (*Legionella pneumophila *serogroup 1) using an immunoenzymetric commercial method (Legionella Urinary Antigen; Binax), by PCR from respiratory samples using an in-house PCR technique from the Institute for Medical Microbiology (IMM) in Zurich, Switzerland, or by culture [22,23].

### Statistical analysis

Discrete variables are expressed as counts (percentage) and continuous variables as medians and interquartile ranges (IQR). Frequency comparison was done by chi-square test. Two-group comparison of normally distributed data was performed by Students t-test. For data not normally distributed, the Mann-Whitney-U test was used. To estimate the potential clinical relevance of laboratory and clinical parameters to diagnose *Legionella *CAP, we used likelihood-ratio tests and univariate and multivariate logistic regression models. Thereby, outcomes were either *Legionella *CAP or non-*Legionella *CAP. For all independent variables in multivariate analysis, we calculated receiver-operating-characteristics (ROC) with the area under the ROC curve (AUC) being an overall diagnostic measure. For all calculations we used STATA 9.2 statistical software (Stata Corp, College Station, Tex). All testing was two-tailed and P-values less than 0.05 were considered to indicate statistical significance.

## Results

### Baseline parameters

This pooled analysis includes 82 patients with the definite diagnosis of *Legionella *CAP and 368 patients with non-*Legionella *CAP. The median age of the pooled 450 patients was 72 years (IQR 58–81) and 62% were males. Patients with *Legionella *CAP were significantly younger (68 (IQR 49–77) vs 73 (IQR 59–82), p = 0.01), and had less frequent underlying chronic obstructive pulmonary disease (10% vs 24%, p = 0.004) and heart disease (24% vs 42%, p = 0.003). Cough (70% vs 89%, p < 0.001), increase in sputum production (34% vs 72%, p < 0.001) and dyspnea (60% vs 72%, p < 0.03), the cardinal symptoms of CAP, were more frequently found in non-*Legionella *CAP, while *Legionella *CAP patients had a higher median body temperature on presentation (39.3°C vs. 38.4°C, p < 0.001). As demonstrated in Table 1, *Legionella *CAP patients had higher concentrations of CRP, lactate dehydrogenase (LDH) and creatinine kinase (CK), more frequently elevated liver enzymes, proteinuria and hemoglobinuria and lower concentrations of sodium, platelets and pH as compared to non-*Legionella *CAP patients. Additional demographic, clinical and laboratory data are presented in Table 1. Cough, increase of sputum production, dyspnea and confusion in patients with *Legionella *CAP were available in 96% (n = 79), 94% (n = 77), 94% (n = 77) and 96% (n = 79), respectively.

**Table 1:** Baseline Characteristics of the 450 Patients with CAP separated by *Legionella *CAP (n = 82) and non-*Legionella *CAP (n = 368).

	***Legionella *CAP ** (n = 82)	**Non-*Legionella *CAP ** (n = 368)	***P-value***
**Demographic characteristics**			
- Age (years)*	68 (49–77)	73 (59–82)	*0.01*
- Sex (male) – no. (%)	57 (70)	221 (60)	*0.11*
**Coexisting illnesses **– no. (%)			
- Congestive heart disease	20 (24)	156 (42)	*0.003*
- Cerebrovascular disease	7 (9)	20 (5)	*0.29*
- Renal dysfunction	23 (28)	100 (27)	*0.15*
- COPD^a^	8 (10)	90 (24)	*0.004*
- Neoplastic disease	17 (21)	53 (14)	*0.15*
**Clinical History **– no. (%)			
- Antibiotic pretreatment	27 (33)	75 (20)	*0.01*
- Cough	55 (70)	329 (89)	*<0.001*
- increase in sputum production	26 (34)	265 (72)	*<0.001*
- Dyspnea	46 (60)	266 (72)	*0.03*
**Clinical and laboratory findings**			
- Confusion – no. (%)	9 (11)	31 (8)	*0.4*
- Respiratory rate (breaths/minute)*	18 (16–28)	22 (20–28)	*0.01*
- Systolic blood pressure (mmHg)*	130 (113–145)	130 (113–143)	*0.9*
- Heart rate (beats/minute)*	100 (86–114)	95 (80–110)	*0.05*
- Body temperature (°C)*	39.3 (38.5–40.1)	38.4 (37.6–39.2)	*<0.001*
**Laboratory findings**			
- C-reactive protein (mg/L)*	241 (171–315)	134 (67–217)	*<0.001*
- Procalcitonin (μg/L)*	2.90 (0.93–6.43)	0.57 (0.19–2.61)	*<0.01*
- Hematocrit (%)*	37 (32–41)	38 (35–40)	*0.17*
- Leucocyte counts (×109/L)*	12.9 (9.5–15.5)	12.8 (8.9–16.8)	*0.47*
- Platelet counts (×109/L)*	191 (141–253)	243 (190–326)	*<0.001*
- Sodium (mmol/L)*	132 (128–135)	136 (133–138)	*<0.001*
< 131 mmol/L – no. (%)	36 (46)	49 (14)	*<0.001*
- Creatinine (umol/L)*	101 (80–144)	105 (83–138)	*0.29*
- Urea (mmol/L)*	8.3 (5.4–14.2)	6.9 (4.9–11.5)	*0.93*
- Elevated liver enzymes^b ^– no. (%)	52 (63)	126 (34)	*<0.001*
- Creatinine kinase (U/L)*	103 (48–417)	77 (41–174)	*<0.01*
- Lactate dehydrogenase (U/L)*	252 (204–382)	214 (175–268)	*<0.001*
- Glucose (mmol/L)*	6.9 (6.1–9.7)	6.8 (5.9–8.4)	*0.12*
- Oxygen saturation (%)*	93 (88–95)	93 (89–96)	*0.46*
- PaO2 (kPa)*	7.7 (7.1–9.9)	8.0 (6.8–9.5)	*0.82*
- pH*	7.46 (7.38–7.49)	7.43 (7.39–7.46)	*0.02*
- Hemoglobinuria – no. (%)	45 (70)	114 (45)	*<0.001*
- Proteinuria – no. (%)	46 (73)	124 (48)	*<0.001*

**Risk assessment**			
- PSI^c ^Points*	117 (90–140)	95 (73–121)	<0.001
- CURB65*	2 (1–2)	1 (1–2)	0.09

**Outcome **– no. (%)			
- Death	13 (16)	33 (9)	0.06
- Admission to ICU^d^	34 (41)	43 (12)	0.01
- Length of hospital stay (days)*	13 (8–20)	11 (6–17)	0.02

* denotes median (interquartile range); ^a ^COPD denotes chronic obstructive pulmonary disease; ^b ^elevated liver enzymes denotes elevation of ASAT and/or ALAT; ^c ^PSI denotes Pneumonia Severity Index; ^d ^ICU denotes intensive care unit.

The diagnosis of the 82 patients with *Legionella *CAP was established with a total of 164 microbiological tests including 72 urinary antigen tests, 54 cultures and 38 PCRs of respiratory specimen. Because in most patients multiple diagnostic tests were performed, addition did not sum up to 100%. As demonstrated in Table 2, three different etiologic groups of *Legionella *were identified, namely 69.5% (n = 59) with only *Legionella pneumophila *serogroup 1, 15.9% (n = 13) with *Legionella pneumophila *and *Legionella *species and 14.6% (n = 12) with only *Legionella *species, whereas in the previous two groups (n = 13 and n= 12) serogroups and species of *Legionella *were not otherwise specified. Among patients who received urinary antigen testing, results for *Legionella pneumophila *serogroup 1 were positive in 59 of 72 patients (82%). In patients with negative urinary antigen testing (n = 13), diagnosis of *Legionella *was established by culture in 5 patients and/or PCR in 13 patients. In patients with no urine antigen testing (n = 10) diagnosis was ascertained by culture and/or PCR. In the 368 patients with non-*Legionella *CAP urinary antigen testing for *Legionella pneumophila *serogroup 1 was performed with negative results. In these patients a causative microorganism in blood was found in 8.4% (*Streptococcus pneumoniae *(6.0%), *Staphylococcus aureus *(0.8%), *Escherichia coli *(0.4%), *Klebsiella pneumoniae *(0.2%) and others (1.0%)).

**Table 2:** Results of microbiological tests (n = 164) in all patients with *Legionella *CAP (n = 82).

**Established by \Etiologic Diagnosis**		***L. pn *1**^**a **^ (n = 57)	***L. pn*^**b **^& L.** **spp.**^**c**^ (n = 13)	***L*. spp** (n = 12)
Urinary antigen testing (n = 72)	Positive (n = 59)	50	9	0
	Negative (n = 13)	1	4	8

Respiratory specimen culture (n = 54)	Positive (n = 23)	14	8	1
	Negative (n = 31)	19	5	7

Respiratory specimen PCR^d ^(n = 38)	Positive (n = 34)	10	13	11
	Negative (n = 4)	4	0	0

^a ^*L. pn *1, ^b ^*L. pn *and ^c ^*L. spp*. denote *Legionella pneumophila *serogroup 1, *Legionella pneumophila *and *Legionella *species; ^d ^PCR denotes polymerase chain-reaction, respectively.

The goldstandard for diagnosis of *Legionella *CAP was the clinical diagnosis of a community-acquired pneumonia with an infiltrate on chest X-ray and at least one positive microbiological test for *Legionella *(urinary antigen testing, culture results from respiratory specimen or PCR from respiratory specimen).

### Diagnostic reliability of clinical and laboratory parameters

**Figure 1:**
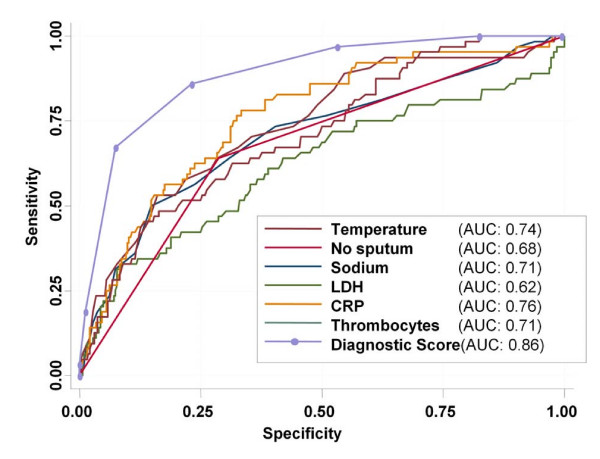
**Receiver Operating Curve (ROC) analysis of different clinical and laboratory parameters to differentiate *Legionella *CAP from non-*Legionella *CAP**. AUC denotes area under the curve; LDH and CRP denote lactate dehydrogenase and C-reactive protein.

**Figure 2:**
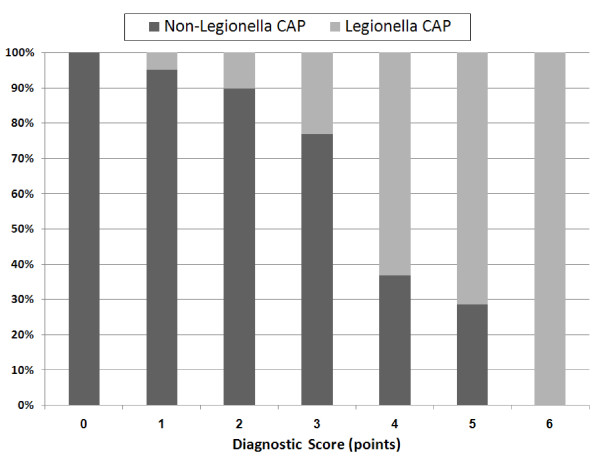
**Patients with non-*Legionella *CAP (dark gray) and *Legionella *CAP (light gray) according to the points of the diagnostic score**. Numbers in the boxes represent total number of patients.

To assess the diagnostic reliability of laboratory parameters and clinical signs to identify *Legionella *CAP patients, we calculated univariate logistic regression analysis (Table 3) with all parameters that were significantly different between *Legionella *and non-*Legionella *CAP patients on presentation. Further, to identify independent predictors of *Legionella*, we calculated multivariate logistic regression analysis (Table 3). Multivariate analysis identified two clinical parameters, namely body temperature (OR 1.67, p < 0.0001), and absence of sputum production (OR 3.67, p < 0.0001), and four laboratory parameters, namely sodium (OR 0.89, p = 0.01), LDH (OR 1.003, p = 0.007), CRP (OR 1.006, p < 0.0001) and platelet counts (OR 0.991, p < 0.0001) as independent predictors of *Legionella *CAP, respectively. To estimate the clinical usefulness of these parameters and to compare sensitivities and specificities, ROC curves using these 6 predictors were calculated (Figure 1). The area under the ROC curve, optimal cut off values and corresponding sensitivities and specificities are presented in table 4 for each variable at the optimal cut off point are presented in Table 4. Using these optimal cut off values, we calculated a 6 point diagnostic score giving a point for each variable beyond the optimal cut off. Patients with *Legionella *CAP had a significantly higher median score as compared to patients without *Legionella *(4 (IQR 3–4) vs 2 (IQR 1–2), p < 0.0001). The odds ratio of this score to predict *Legionella *was 3.34 (95%CI 2.57–4.33, p < 0.0001). With an area under the curve (AUC) of 0.86 (95%CI 0.81–0.90), the diagnostic accuracy of this diagnostic score was significantly better as compared to each single one of the six parameters. Table 5 shows corresponding sensitivities, specificities and the number of patients with and without *Legionella *CAP for each cut off point of the diagnostic score. In Figure 2 the distribution of *Legionella *and non-*Legionella *CAP according to the score is demonstrated. Of the 191 patients (42%) with 0 or 1 point, only 6 (3%) had *Legionella *pneumonia. Conversely, of the 73 patients (16%) with ≥4 points, 48 patients (66%) had *Legionella *CAP.

**Table 3:** Prediction of *Legionella *CAP (n = 82) in univariate (a) and multivariate (b) logistic regression analysis

**Univariate analysis**				
**Predictor**	**Odds ratio**	(95% CI*)		**P-value**
Age	0.98	0.97	1.00	0.017
Temperature	1.93	1.51	2.46	<0.0001
No dyspnea	1.75	1.06	2.9	0.03
No cough	3.7	2.05	6.6	<0.0001
No sputum	5.14	3.04	8.7	<0.0001
Sodium	0.86	0.82	0.91	<0.0001
Elevated liver enzymes^1^	3.32	2.02	5.48	<0.0001
Lactate dehydrogenase	1.003	1.002	1.005	0.001
Creatinine kinase	1.001	1.000	1.001	0.001
C-reactive protein (CRP)	1.006	1.004	1.008	<0.0001
Platelet counts	0.993	0.990	0.996	<0.0001
Hemoglobinuria	2.95	1.64	5.32	<0.0001
Proteinuria	2.88	1.56	5.29	0.001

**Multivariate analysis**				

**Predictor**	**Odds ratio**	**(95% CI)**		**P-value**
Temperature	1.67	1.23	2.32	<0.0001
No sputum	3.67	1.8	7.4	<0.0001
Sodium	0.89	0.84	0.96	0.01
Lactate dehydrogenase	1.003	1.000	1.005	0.007
C-reactive protein (CRP)	1.006	1.003	1.009	<0.0001
Platelet counts	0.991	0.987	0.995	<0.0001

* Cl denotes confidence interval; ^1 ^elevated liver enzymes denotes ASAT and/or ALAT.

**Table 4:** Area under the curve (AUC) of receiver operating curve (ROC) characteristic plot analysis.

**Parameter**	**AUC**	95% CI*	***P-value***	**Optimal cut off**	**Sensitivity**	**Specificity**
**Combined Score**	0.86	0.81–0.90	^-^	< 2	78.0	78.8

**Temperature**	0.74	0.63–0.78	*<0.0001*	> 39.4	48.1	84.4
**No sputum**	0.68	0.61–0.74	*<0.0001*	*-*	*-*	*-*
**Sodium**	0.71	0.63–0.78	*<0.0001*	< 133	64.6	70.8
**Lactate dehydrogenase**	0.62	0.53–0.71	*<0.0001*	> 225	67.1	58.1
**C-reactive protein**	0.76	0.70–0.82	*<0.0001*	> 187	71.6	64.7
**Platelet counts**	0.71	0.64–0.78	*<0.0001*	< 171	45.7	83.6

*CI denotes confidence interval;

**Table 5:** Diagnostic accuracy of the diagnostic score at different cut off points.

**Points**	**Sensitivity**	**Specificity**	***Legionella *CAP**	**Non-*Legionella *CAP**
≥ 0	100	*-*	0 (0%)	70 (19.0%)
≥ 1	92.7	50.3	6 (7.3%)	115 (31.3%)
≥ 2	78.0	78.8	12 (14.6%)	105 (28.5%)
≥ 3	58.5	93.2	16 (19.5%)	53 (14.4%)
≥ 4	14.6	98.9	36 (43.9%)	21 (5.7%)
≥ 5	2.4	100	10 (12.2%)	4 (1.1%)
6	*-*	100	2 (2.4%)	0 (0%)

## Discussion

The timely identification of *Legionella *in patients presenting with CAP to the emergency department is challenging because single clinical and laboratory parameters have shown low sensitivity and/or specificity. A reliable diagnostic score integrating different parameters is lacking [8]. The results of this study indicate that six clinical and laboratory parameters, namely high body temperature, absence of sputum production, low serum sodium and platelet counts, and high LDH and CRP concentrations combined in a diagnostic score reliably estimate the likelihood of *Legionella *in patients who present with CAP to the emergency department.

Current guidelines on the management of patients with CAP recommend that empirical antibiotic coverage should be extended to *Legionella *in "suspicious cases", although no single parameter can reliably identify or rule out patients at risk for *Legionella *[8,13]. Previous studies have addressed this dilemma and assessed potential predictors of *Legionella *CAP in different settings [1,5-12]. A Spanish study found that *Legionella *should be suspected in middle-aged, alcohol drinking, healthy male patients, if patients lack response to previous beta-lactamic drugs, if headache, diarrhea, severe hyponatremia, and elevation in serum CK levels were present, or if no cough, expectoration, and thoracic pain were found [12]. A recent critical review included 13 different studies that reported clinical details for the evaluation of *Legionella *CAP, however, concluded that using a syndromic approach cannot be recommended and, thus, an empiric antibiotic therapy covering for *Legionella *for all patients with CAP requiring hospitalization should be recommended [8]. This, however, results in indiscriminate use of, mostly unnecessary, dual antibiotic therapy and thus, increased antibiotic exposure and emergence of resistance. Two previous studies have proposed a clinical prediction rule to identify *Legionella *CAP [5,6]. The CBPIS score has been proposed with a maximum of 17 points based on the evaluation of temperature, serum creatinine, sodium and LDH concentrations, headache, vomiting and smoking. However, in a prospective validation study, this scoring system had a low sensitivity and/or specificity to diagnose or exclude *Legionella *CAP [5]. Guidance of antibiotic therapy using this score would have left half of the patients with *Legionella *CAP without specific coverage and would have led to the administration of an unnecessarily broad antibiotic regimen in 14% of patients without *Legionella*. In addition, the model was unable to distinguish between *Legionella pneumophila *and *Streptococcus pneumoniae*, because the majority of patients were categorized into the "intermediate-probability" group. Similarly, the clinical criteria proposed by the Winthrop-University Hospital (WUH) for the identification of Legionellosis showed an inadequately low sensitivity and specificity of 78% [6]. In addition, the WUH study included clinical data collected throughout the first 7 days of hospitalization limiting its use as a screening tool in an emergency department setting. Importantly, both studies based their scoring system on the comparison of *Legionella *CAP with pneumococcal CAP, which does not reflect the clinical situation in the emergency department, where the decision about empirical antibiotic therapy must be taken in all patients with CAP. In this study, a thorough and comprehensive 10 year retrospective data collection was conducted and data from two prospective studies presenting to the emergency department of the same institution were used to compare clinical and laboratory parameters. Thus we believe the proposed score reflects the clinical situation more realistically.

Simplicity of a diagnostic score is a major determinant for its future usefulness in daily practice. The WUH scale consists of 23 different criteria, which increases its complexity and may correlate with non-adherence as observed in clinical severity prediction scores [6,24]. The herein proposed scoring system composes of only 6 routinely measured clinical and laboratory parameters that showed an independent predictive value in multivariate logistic regression analysis and a high overall diagnostic performance as demonstrated in ROC analysis.

As with any diagnostic score, the "optimal" cut off should be chosen based on the pretest probability and the clinical context, particularly the risk for adverse medical outcomes of patients. Supported by recent guidelines, treatment decision on the empirical antibiotic therapy should be based on both, the diagnostic probability and the risk of patients based on a prognostic assessment. Importantly, the PSI – but not the CURB65 score – showed a high prognostic accuracy to predict adverse medical outcomes in patients with *Legionella *CAP in this study. This finding is reassuring because prognostic CAP scores mainly depend on age and *Legionella *CAP patients tend to be younger. Thus, taking the diagnostic limitations of urinary antigen testing into account, empirical antibiotic therapy covering for atypical pathogens in patients with negative urinary antigen testing but high diagnostic probability (diagnostic score ≥ 4) may be advisable. Conversely, in low risk patients with low diagnostic probability (diagnostic score ≤ 1) and no clinical or diagnostic evidence of CAP caused by other atypical pathogens, macrolides may initially be withheld until the results of the urinary antigen tests become available.

Some limitations should be considered in interpreting our results. With a retrospective design, our results are preliminary and prospective validation is needed prior to a widespread use in clinical practice. The diagnosis of *Legionella *spp. in non-*Legionella *CAP patients was performed routinely by urinary antigen testing without PCR, culture or serology and we may have classified some patients incorrectly. Moreover there was no further routinely investigation for other atypical bacterial pathogens. We chose the 10 year retrospective design to provide the necessary number of *Legionella *cases to calculate multivariate analysis with adequate power. The proposed score consists of mainly objectively measurable parameters, which may robustly be ascertained in retrospect. Still, anamnestic parameters (i.e. absence of sputum production), even if highly available, may not be accurately assessed in retrospect and are thus underestimated in favour of laboratory parameters and clinical data.

## Conclusion

In conclusion, the results of this study suggest that six clinical and laboratory signs embedded in a simple diagnostic score allow better differentiation of *Legionella *from unselected patients with non-*Legionella *CAP presenting to the emergency department. If confirmed in prospective studies, this score might improve the timing and choice of empirical antibiotic therapy and thus reduce the associated morbidity and mortality and the emergence of bacterial resistance.

## Competing interests

The authors declare that they have no competing interests.

## Authors' contributions

PS, BM, RF, RZ, and JH had the idea for the study and directed the study design. PS, BM, MCC, RF, RZ, JH, AT, RF and IS directed the data collection. PS RF, RZ and JH analyzed the data and wrote the first report. PS, BM, MCC, RF, RZ, JH, AT, RF and IS had substantial contributions in planning of the study, data collection, interpretation of data and/or writing of the manuscript.

## Pre-publication history

The pre-publication history for this paper can be accessed here:


